# Efficacy of 24-weekly vs 12-weekly decapeptyl SR treatment in central precocious puberty: a UK multicentre retrospective cohort study

**DOI:** 10.1210/clinem/dgag096

**Published:** 2026-03-06

**Authors:** Rachel Varughese, Lydia Lake, Bharathy Kothayan, Nathalie S Ahmed, Katherine Aitken, Karishma Bhavsar, Joe Edwards, Angel Newsome, Sofia P S Pinto, David Thomas, Amy Woodhouse, Julia Russell, Jacqueline P O’Sullivan, Emily Connolly, Isabel Sharratt, Tim Cheetham, Charlotte J Elder, Claire L Wood, Sasha R Howard

**Affiliations:** Centre for Endocrinology, William Harvey Research Institute, QMUL, London EC1M 6BQ, UK; Department of Paediatric Endocrinology, Royal London Children's Hospital, Barts Health NHS Trust, London E1 1BB, UK; Centre for Endocrinology, William Harvey Research Institute, QMUL, London EC1M 6BQ, UK; Centre for Endocrinology, William Harvey Research Institute, QMUL, London EC1M 6BQ, UK; Centre for Endocrinology, William Harvey Research Institute, QMUL, London EC1M 6BQ, UK; Department of Paediatric Endocrinology, Royal Victoria Infirmary, Newcastle Hospitals NHS Foundation Trust, Newcastle upon Tyne NE1 4LP, UK; Centre for Endocrinology, William Harvey Research Institute, QMUL, London EC1M 6BQ, UK; Department of Paediatric Endocrinology, Royal Victoria Infirmary, Newcastle Hospitals NHS Foundation Trust, Newcastle upon Tyne NE1 4LP, UK; Translational and Clinical Research Institute, Newcastle University, Newcastle upon Tyne NE1 7RU, UK; Centre for Endocrinology, William Harvey Research Institute, QMUL, London EC1M 6BQ, UK; Centre for Endocrinology, William Harvey Research Institute, QMUL, London EC1M 6BQ, UK; Department of Paediatric Endocrinology, Sheffield Children's NHS Foundation Trust, Sheffield S10 2TH, UK; Department of Paediatric Endocrinology, Sheffield Children's NHS Foundation Trust, Sheffield S10 2TH, UK; Department of Paediatric Endocrinology, Royal Victoria Infirmary, Newcastle Hospitals NHS Foundation Trust, Newcastle upon Tyne NE1 4LP, UK; Department of Paediatric Endocrinology, Royal Victoria Infirmary, Newcastle Hospitals NHS Foundation Trust, Newcastle upon Tyne NE1 4LP, UK; Department of Paediatric Endocrinology, Royal London Children's Hospital, Barts Health NHS Trust, London E1 1BB, UK; Department of Paediatric Endocrinology, Royal London Children's Hospital, Barts Health NHS Trust, London E1 1BB, UK; Department of Paediatric Endocrinology, Royal Victoria Infirmary, Newcastle Hospitals NHS Foundation Trust, Newcastle upon Tyne NE1 4LP, UK; Translational and Clinical Research Institute, Newcastle University, Newcastle upon Tyne NE1 7RU, UK; Department of Paediatric Endocrinology, Sheffield Children's NHS Foundation Trust, Sheffield S10 2TH, UK; Department of Clinical Medicine, School of Medicine and Population Health, Faculty of Medicine, Dentistry and Health, University of Sheffield, Sheffield S10 2RX, UK; Department of Paediatric Endocrinology, Royal Victoria Infirmary, Newcastle Hospitals NHS Foundation Trust, Newcastle upon Tyne NE1 4LP, UK; Translational and Clinical Research Institute, Newcastle University, Newcastle upon Tyne NE1 7RU, UK; Centre for Endocrinology, William Harvey Research Institute, QMUL, London EC1M 6BQ, UK; Department of Paediatric Endocrinology, Royal London Children's Hospital, Barts Health NHS Trust, London E1 1BB, UK

**Keywords:** central precocious puberty, decapeptyl, triptorelin, GnRHa

## Abstract

**Objective:**

To evaluate the efficacy of 24-weekly decapeptyl SR (Triptorelin) treatment, compared with the 12-weekly regimen, in children with central precocious puberty (CPP).

**Methods:**

A multicenter retrospective cohort study was conducted on patients with CPP treated with gonadotropin-releasing hormone analog (GnRHa) therapy between September 2008 and December 2024. Participants were recruited from 3 tertiary pediatric endocrinology centers in the United Kingdom: the Royal London Hospital (Barts Health NHS Trust), Sheffield Children's Hospital (Sheffield Children's NHS Foundation Trust), and the Royal Victoria Infirmary (Newcastle upon Tyne Hospitals NHS Foundation Trust). Patients received either Decapeptyl SR 11.25 mg every 12 weeks or 22.5 mg every 24 weeks. Clinical, biochemical, and radiological data were collected at baseline and follow-up to assess hypothalamic–pituitary–gonadal (HPG) axis suppression and pubertal progression.

**Results:**

Of 247 patients reviewed (220 girls and 27 boys), 164 were eligible for analysis: 69 in Group 1 (12 weekly) and 95 in Group 2 (24 weekly). Both regimens achieved effective HPG axis suppression, with no significant differences in luteinizing hormone, follicle-stimulating hormone, or sex steroid concentrations. Clinical outcomes, including height velocity, body mass index, and Tanner staging, were comparable. The 24-weekly preparation was well-tolerated and demonstrated equivalent suppression of pubertal progression. Among patients who expressed a preference, all favored the 24-weekly schedule.

**Conclusion:**

This UK multicenter study provides evidence that 24-weekly Decapeptyl SR is both efficacious and well-tolerated for the management of CPP, with comparable outcomes to the standard 12-weekly regimen. This is the first comparative analysis to establish clinical equivalence between these 2 regimens, which is particularly important given the reduced frequency of clinical monitoring in the longer-acting preparation. Fewer injections may improve adherence and patient satisfaction, alongside potential cost savings.

Central precocious puberty (CPP) refers to the premature onset of concordant pubertal development, resulting from early activation of the hypothalamic–pituitary–gonadal (HPG) axis. The definition of precocious puberty is based on age thresholds derived from population data reflecting normal pubertal onset and is essential for distinguishing early but normal variation from true pathological precocity. In girls, CPP is clinically defined by the development of breast buds (thelarche or Tanner stage B2) before the age of 8 years, and in boys by a testicular volume ≥4 mL (Tanner stage G2) before the age of 9 years (1). The condition exhibits a marked female predominance, with a reported female-to-male ratio ranging from 10:1 to 20:1 (2, 3). Additional secondary sexual characteristics may be observed, including the development of pubic and axillary hair, accelerated linear growth, behavioral or mood changes, and premature menarche in girls.

The premature activation of the HPG axis leads to elevated levels of circulating gonadotropins; luteinizing hormone (LH) and follicle-stimulating hormone (FSH) and also sex steroid hormones; estradiol in girls and testosterone in boys. Estradiol plays a central role in endochondral ossification at the growth plate, driving longitudinal bone growth during childhood in both sexes, with testosterone in boys converted to estradiol by aromatization. In CPP, elevated estradiol concentrations at an earlier age result in initially rapid growth and advanced bone age (BA) but ultimately lead to premature epiphyseal closure and reduced adult height (4). Beyond physical consequences, the psychosocial impact of early puberty may be significant. Girls experiencing early menarche are at increased risk of emotional distress, depression, anxiety, and behavioral issues, with some effects persisting into adulthood (5). Evidence also suggests an association between early menarche and premature natural menopause (6).

While CPP can result from various etiologies, the most common forms are idiopathic or genetically driven, with pathogenic variants in a small number of genes identified in many familial cases (7, 8). Although rare, organic central nervous system pathologies are more frequently observed in boys with CPP (9, 10), while the vast majority of CPP in girls is idiopathic. Globally, the incidence of CPP is rising, placing increased demand on pediatric endocrinology services for timely diagnosis and management (11, 12).

The current standard of care for children with CPP involves suppression of the HPG axis using extended-release gonadotropin-releasing hormone analogs (GnRHa) (13). These agents produce sustained stimulation of the GnRH receptor, overriding physiological GnRH pulsatility, leading to receptor desensitization. There is consequent reduced secretion of biologically active LH and FSH and a relative increase in secretion of the free α-subunit rather than intact gonadotropins (14, 15). Various preparations are available internationally, although some are not currently licensed in the UK, including 1-, 3-, and 6-monthly leuprolide acetate depots and the long-acting 12-month subcutaneous histrelin acetate implant (16, 17). The most commonly prescribed medication in the UK is Decapeptyl SR (Triptorelin pamoate), traditionally prescribed as the 11.25 mg regimen, administered intramuscularly every 12 weeks (18). Treatment aims to halt or reverse pubertal progression, prevent menstruation in girls, and preserve adult height potential. These goals are critical for mitigating both the physical and psychological impacts of CPP.

Despite the efficacy and safety of the 12-weekly regimen, several limitations remain including injection site pain, inflammatory reactions, financial costs of frequent dosing, and the psychosocial burden of repeated hospital visits and school absences. To address these issues, longer-acting formulations have been developed. Decapeptyl SR, administered as a 24-weekly 22.5 mg depot injection (Triptorelin embonate), has been used increasingly in the UK since an international trial of 44 children with CPP demonstrated safety and efficacy for this preparation (19). However, while publications have explored the use of the 24-weekly preparation in children with CPP (19-21), there is no published literature to demonstrate its efficacy or tolerability as compared to the 12-weekly preparation. Potential benefits of a sustained-release formulation include more patient-friendly schedules, as compared to monthly or quarterly injections, which may be associated with higher treatment adherence and reduced financial cost of hospital appointments and specialist nursing time. However, their adoption in routine practice has been cautious, reflecting ongoing clinical uncertainty regarding the consistency of pubertal suppression and reduced opportunities for monitoring, highlighting the need for comparative real-world evidence.

This study aimed to evaluate the effectiveness of the 24-weekly GnRH analog formulation as compared to the standard 12-weekly regimen in patients with nonorganic CPP, across 3 large tertiary pediatric endocrine centers in the UK. Pubertal and auxological outcomes were compared between patients treated with 12-weekly or 24-weekly formulations of Decapeptyl SR over a 2-year follow-up period.

## Methods

### Study design and participants

A multicenter retrospective observational cohort study was conducted for patients diagnosed with CPP and treated with the GnRHa Decapeptyl SR, between September 2008 and December 2024. Patients were recruited from 3 UK pediatric endocrine centers: Royal London Hospital (RLH), Barts Health NHS Trust, Royal Victoria Infirmary (RVI), Newcastle upon Tyne Hospitals NHS Foundation Trust, and Sheffield Children's Hospital (SCH), and Sheffield Children's NHS Foundation Trust. The study was registered and approved as a service evaluation with all 3 participating trusts and conducted according to STROBE guidelines. Anonymized data were shared between centers in accordance with local governance approvals and institutional data-sharing agreements.

Patients were identified through hospital electronic medical records. Patients were eligible for inclusion if they had received Decapeptyl SR therapy between September 2008 and December 2024. Girls were eligible for inclusion if they exhibited progressive pubertal development at more than one clinical assessment at least 3 months apart, defined as Tanner breast stage 2 (B2) or above, before the age of 8 years, or menarche before 10 years. Boys were included if they had testicular volume ≥4 mL (Tanner genital stage 2 or above) before the age of 9 years. All patients required biochemical confirmation of HPG axis activation, demonstrated by either basal LH >0.5 IU/L or GnRH-stimulated LH >5 IU/L after a standard GnRH stimulation test (22). Patients were excluded if they had a diagnosis of peripheral precocious puberty or an identified organic or syndromic cause of CPP (including a CNS lesion detected on magnetic resonance imaging [MRI] brain).

Outcomes included the biochemical suppression of the HPG axis, based on LH, FSH, and sex steroid concentrations and clinical markers of puberty, including height velocity (HV), body mass index (BMI), and Tanner staging.

Comprehensive data were extracted from electronic patient records at baseline, 6 months, 1 year, 2 years, and (if applicable) treatment endpoint. Data included demographic and clinical information, pubertal staging, anthropometric measurements, biochemical markers, and BA assessments.

### Treatment arms

Participants were categorized into 2 groups: those in Group 1 (12-weekly) had received 11.25 mg every 10 to 12 weeks, and participants in Group 2 (24-weekly) had received 22.5 mg every 22 to 24 weeks. Individuals treated at any other dosing intervals were excluded.

Patients had either completed treatment or, for those still undergoing treatment, had been treated with Decapeptyl for a minimum of 12 months.

### Auxological assessment

Auxological parameters were assessed at baseline pretreatment and on treatment at 6 months, 1 year, and 2 years. For height standard deviation score (SDS) data, datasets were normally distributed in certain groups and nonnormally distributed in others. Therefore, all height SDS data were treated as nonparametric. HV data were parametric. Near-adult height data were available for a subset of patients treated at RLH who had reached near–adult height, defined as growth velocity <2 cm/year following cessation of GnRHa therapy and completion of pubertal progression. Heights are presented as height SDS. Patients with conditions known to independently affect growth (including significant scoliosis or eating disorders) were excluded from this analysis, as well as one deceased patient.

### Pubertal assessment

Pubertal parameters were assessed at baseline (pretreatment) and on treatment at 6 months, 1 year, and 2 years. Physical pubertal staging was recorded using Tanner criteria. In boys, testicular volume was measured with a Prader orchidometer; in girls, breast development was assessed via inspection/palpation. All patients were also evaluated for axillary and pubic hair development and other physical signs of puberty. Height, weight, BMI, and HV SDS were tracked over time. BMI centiles were calculated using UK-WHO growth standards (23). BA was assessed from left hand and wrist radiographs using BoneXpert with either the Tanner-Whitehouse 3 (TW3) or Greulich and Pyle (GP) method. HV and BA–chronological age difference (BA–CA) were analyzed at baseline and 2 years only, due to limited interim data.

### Biochemical monitoring

Key hormonal markers, including serum LH, FSH, estradiol (in girls) and testosterone (in boys), were measured at baseline and monitored throughout treatment at the clinician's discretion. Prolactin, thyroid function tests, and MRI brain scans were performed in all patients at diagnosis to rule out alternative etiologies. HPG-axis suppression was defined as a basal LH <1 IU/L measured immediately prior to the next scheduled Decapeptyl injection (24). The LH laboratory assay used was electrochemiluminescence immunoassay—ECLIA in all 3 centers.

### Statistical analysis

All data were analyzed using GraphPad Prism (version 10.4.0). Normality of continuous variables was assessed using the Shapiro–Wilk test. For comparisons between 2 groups, unpaired *t*-tests with Welch's correction were used for parametric data (HV) and Mann–Whitney U tests for non-parametric data (height SDS, BMI SDS, Tanner stage, biochemical markers, BA). Effect sizes were reported as mean differences with 95% confidence intervals (Welch's t-test) or Hodges–Lehmann (HL) median differences with 95% confidence intervals (Mann–Whitney U test). A *P*-value of <.05 was considered statistically significant. For proportions (eg, LH suppression rates), binomial 95% confidence intervals were calculated for each group. Differences between groups were expressed as risk differences with 95% CIs. Sex-stratified comparative analyses were undertaken where sample size permitted; in boys, limited numbers precluded robust comparative analysis in some outcomes.

## Results

A total of 247 children with CPP were identified across 3 tertiary centers: RLH (*n* & 50), RVI (*n* & 90), and SCH (*n* & 107) (Table 1). Age at diagnosis ranged from 1 to 11 years. Mean age at diagnosis was 7.47 ± 1.50 years for girls and 7.50 ± 1.94 years for boys and was similar across the 3 centers for both girls (*P* = .183) and boys (*P* = .896). The cohort comprised 220 girls and 27 boys. Of these, 164 patients met inclusion criteria for posttreatment analysis, with 69 receiving 12-weekly Decapeptyl SR (Group 1, girls = 62, boys = 7) and 95 receiving 24-weekly Decapeptyl SR (Group 2, girls = 86, boys = 9). Girls constituted the majority of patients in both treatment groups, reflecting the known epidemiology of CPP; boys represented a smaller proportion but were included in analyses where data permitted. Given the small number of boys, formal comparative analyses by treatment regimen were performed primarily in girls, with male data reported descriptively where available.

**Table 1:** Patient characteristics at baseline presentation across centers

	Girls			Boys		
	RLH (*n* = 46)	RVI (*n* = 77)	SCH (*n* = 97)	RLH (*n* = 4)	RVI (*n* = 13)	SCH (*n* = 10)
Age (years)	7.4 ± 1.4	7.3 ± 1.7	7.7 ± 1.4	7.3 ± 2.1	7.8 ± 1.9	7.0 ± 2.3
Height (cm)	135.7 (131.3-141.6)	133.7 (126.8-141.5)	132.1 (125.9-138)	135.9 (132.5-139.3)	136.4 (129.0-146.3)	131.2 (117.7-141.1)
Height SDS	1.8 (1.2-2.5)	1.3 (0.3-2.5)	1.1 (0-1.9)	3.3 (1.3-5.2)	2.4 (1.1-3.2)	0.4 (0-1.4)
Weight (kg)	35.8 (28.9-35.0)	33.0 (26.8-40.6)	31.6 (26.0-37.4)	32.4 (28.7-33.5)	35.0 (29.4-43.1)	31.2 (23.2-53.9)
Weight SDS	1.4 (0.9-2.2)	1.6 (0.4-2.1)	1.4 (0.5-2.4)	2.0 (0.8-3.3)	2.5 (1.1-3.1)	1.1 (0.5-1.3)
BMI (kg/m^2^)	19.2 (16.5-20.9)	18.6 (16.7-20.5)	18.2 (16.4-19.9)	16.5 (16.4-16.7)	18.7 (17.5-21.0)	19.5 (16.9-25.5)
BMI SDS	1.2 (0.1-1.9)	1.2 (0.4-2.0)	1.2 (0.3-2.0)	0.6 (0.4-0.7)	1.2 (0.5-2.7)	1.5 (−0.2 to 1.9)
BA–CA (years)	2.6 ± 1.5	2.6 ± 1.6	1.7 ± 1.2	4.5 ± 2.9	4.0 ± 2.6	1.2 ± 2.1

Normality of the data was assessed using the Shapiro–Wilk test. Age (years) and bone age minus chronological age (BA–CA) were normally distributed and are presented as mean ± standard deviation (SD). All other continuous variables (height, weight, and BMI) were nonnormally distributed and are presented as median (interquartile range).

Abbreviations: BMI, body mass index; BA, bone age, CA, chronological age; RLH, Royal London Hospital; RVI, Royal Victoria Infirmary, Newcastle-Upon-Tyne; SCH, Sheffield Children's Hospital; SDS, standard deviation score.

### Baseline auxology

At baseline (pretreatment), height SDS was similar between Group 1 (12-weekly) and Group 2 (24-weekly) groups for both girls (median 1.45 vs 1.36; 95% CI −0.30 to 0.45, *P* = .92) and boys (1.38 vs 1.38; 95% CI −0.40 to 0.42, *P* = .79). Baseline height, weight, and BMI were also comparable across centers for girls, with greater variability in boys likely due to smaller numbers. BMI SDS did not differ significantly between regimens in either girls (95% CI −0.25 to 0.29, *P* = .83) or boys (95% CI −0.18 to 0.34, *P* = .35). In all 3 cohorts, BA was advanced relative to CA, consistent with CPP. Regional variation was noted in girls, with the SCH cohort showing less BA advancement (BA–CA) at presentation (95% CI −1.4 to −0.4 years, *P* = .001), a difference not observed in boys (95% CI −1.8 to 0.4, *P* = .83).

### Baseline pubertal staging

**Figure 1:**
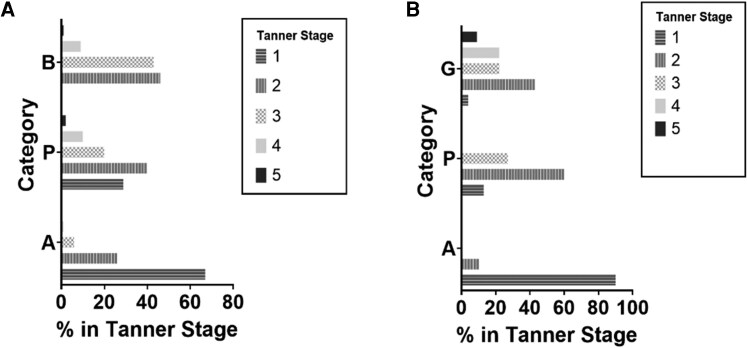
Tanner staging at baseline presentation in (A) girls (*n* = 220) and (B) boys (*n* = 27). Combined percentage of girls or boys in each stage pretreatment, including breast development (B), genital development (G), pubic hair growth (P), and axillary hair growth (A).

Tanner staging at presentation was advanced across all patients, in keeping with a diagnosis of CPP. Due to the retrospective design, data completeness varied across parameters and timepoints; therefore, sample sizes (*n*) are reported for each analysis. For girls, at baseline presentation, 37% were classified as Tanner breast (B) stage 2 (*n* = 81/220), 43% B3 (*n* = 95/220), 18% B4 (*n* = 40/220), and 2% B5 (*n* = 4/220). For pubic hair (P) staging, 27% were stage P1 (*n* = 59/216), 34% P2 (*n* = 73/216), 23% P3 (*n* = 50/216), 13% P4 (*n* = 29/216), and 2% P5 (*n* = 5/216). For axillary hair (A) staging 60% were stage A1 (*n* = 110/184), 32% A2 (*n* = 58/184), and 9% A3 (*n* = 16/184). For boys, at baseline presentation, 0% were Tanner genital (G) stage 1, 41% G2 (*n* = 11/27), 30% G3 (*n* = 8/27), 19% G4 (*n* = 5/27), and 11% G5 (*n* = 3/27). For pubic hair (P) staging, 7% were stage P1 (*n* = 2/27), 48% P2 (*n* = 13/27), 19% P3 (*n* = 5/27), 26% P4 (*n* = 7/27), and 0% P5. For axillary hair (A) staging, 86% were stage A1 (*n* = 12/14), 14% A2 (*n* = 2/14), and 0% A3. The median Tanner stages for girls were B3, P2, A1, and for boys were G2, P2, A1 (Fig. 1A and 1B).

### Treatment regimens

At the end of data collection, 162 patients had completed treatment (RLH: 26 [23 girls, 3 boys]; RVI: 59 [52 girls, 7 boys]; SCH: 77 [73 girls, 4 boys]), 77 patients remained on treatment (RLH: 24 [23 girls, 1 boy]; RVI: 31 [25 girls, 6 boys]; SCH: 22 [18 girls, 4 boys]), and 8 were lost to follow-up [6 SCH girls, 2 SCH boys]. Treatment duration ranged from 12 months to 11 years and 10 months. Median treatment duration was 37.5 months (IQR 27-49) for girls and 49.4 months (IQR 35-64) for boys on 11.25 mg Decapeptyl SR 12-weekly, and 27.2 months (IQR 18-36) for girls and 33.5 months (IQR 22-45) for boys on 22.5 mg 24 weekly.

### Posttreatment outcomes

**Figure 2:**
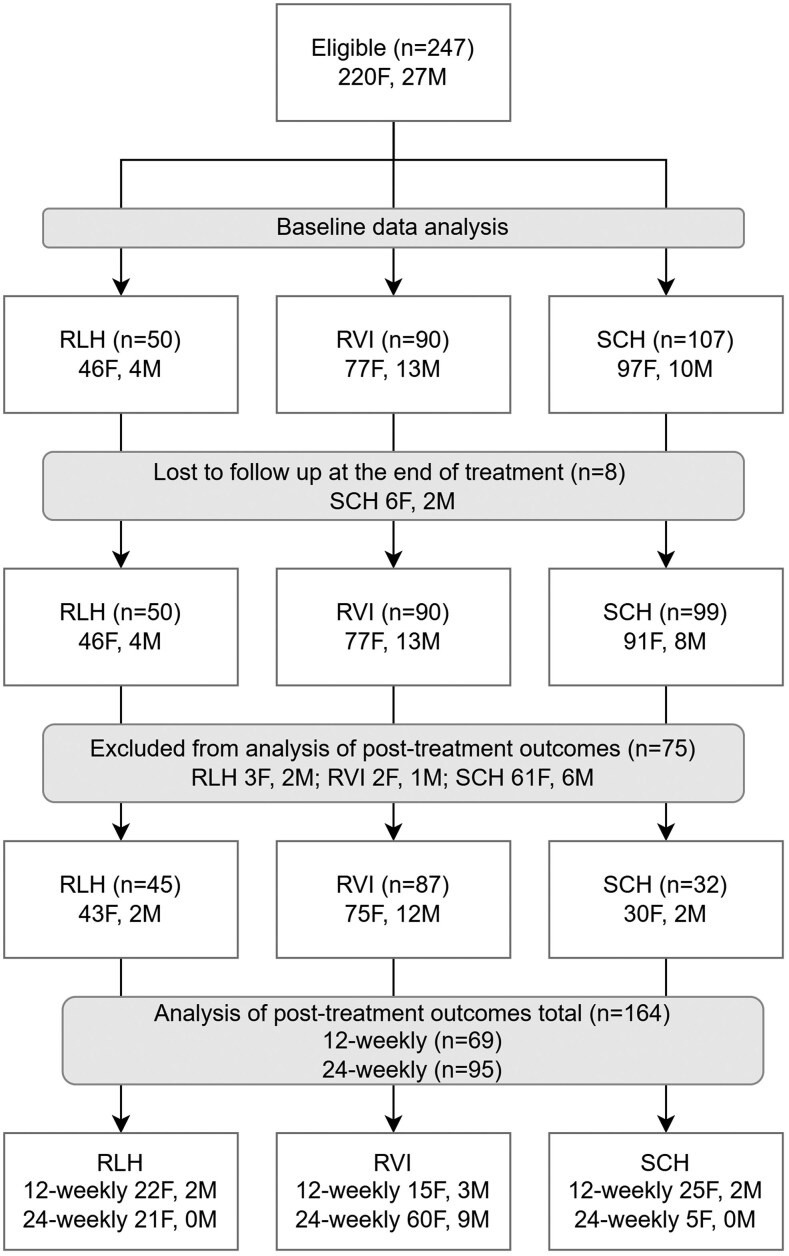
Flow diagram showing participant selection and distribution across cohorts (RLH, RVI, SCH) from eligibility assessment to posttreatment analysis.

A total of 75 patients [5/48 RLH, 3/90 RVI, 67/99 SCH] were excluded from the analysis of posttreatment outcomes, as their treatment trajectories deviated by more than 2 weeks from the standard for that treatment arm (ie, < 10 weekly 11.25 mg and <22 weekly 22.5 mg), precluding appropriate group classification into the 2 predefined treatment regimens (Fig. 2). The remaining 164 patients from RLH (*n* & 45), RVI (*n* & 87) and SCH (*n* & 32) were grouped into 2 treatment categories: Group 1 (12-weekly)—Decapeptyl 11.25 mg, *n* & 69; and Group 2 (24-weekly)—Decapeptyl 22.5 mg, *n* & 95. 118 were treated with these doses at the standard intervals. 46 patients were treated with either 11.25 mg at 10-weekly (*n* & 39) or 22.5 mg 22-weekly (*n* & 7) intervals and were grouped into Group 1 (12 weekly) and Group 2 (24 weekly), respectively. Treatment allocation reflected clinician and family preference and evolving local practice rather than age at diagnosis; use of the 2 regimens was similar in children diagnosed before and after 7 years of age. Among those diagnosed before 7 years, both regimens were commonly used (12 weekly: *n* & 13; 24 weekly: *n* & 17).

### Impact of treatment on auxological parameters

**Figure 3:**
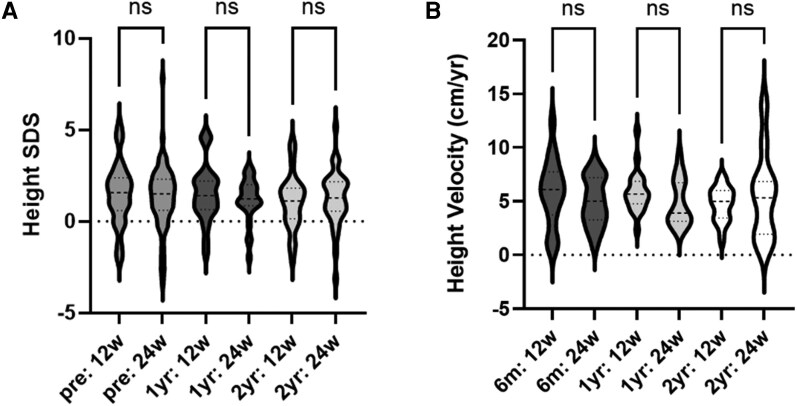
Violin plots demonstrating height SDS and height velocity comparison between 12-weekly and 24-weekly treatment. (A) Height SDS at pretreatment, 1-year, and 2-year timepoints on GnRHa treatment, by treatment group (12 and 24 weeks). Data combined for girls and boys due to the low number of male patients. Height SDS was nonnormally distributed and analyzed using the Mann–Whitney *U* test. (B) Height velocity for girls at 6 months, 1-year, and 2-year timepoints on GnRHa treatment, by treatment group (12- and 24-weekly). Height velocity was normally distributed and analyzed using unpaired *t*-tests with Welch's correction.

Height SDS did not differ significantly between treatment groups at 1 or 2 years. Data from girls and boys were combined due to the low sample size of male data. At 1 year, median values were 1.40 vs 1.23 (Hodges–Lehmann [HL] difference 0.09, 95% CI −0.33 to 0.66; *P* & .66), and at 2 years 1.12 vs 1.28 (−0.16, 95% CI −0.40 to 0.23; *P* & .59) for the Group 1 (12-weekly) and Group 2 (24-weekly), respectively (Fig. 3A). Similarly, no significant differences in HV were observed at 6 months (mean difference −0.89 cm/year, 95% CI −2.77 to 1.00; *P* = .34), 1 year (−1.00 cm/year, −2.38 to 0.38; *P* = .15), or 2 years (+0.80 cm/year, −1.98 to 3.57; *P* = .54) in girls (Fig. 3B). For HV, data for boys in Group 2 (24-weekly) were insufficient for a reliable comparison.

**Figure 4:**
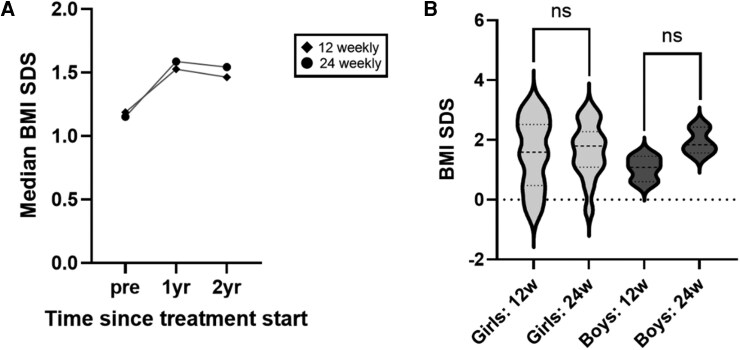
Participant BMI during GnRHa therapy. A Progression of median BMI SDS for girls and boys in Group 1 (12-weekly) (*n* = 40) or 24-weekly (*n* = 88) regimens from baseline to 2 years. B Two years post-GnRHa (Decapeptyl) treatment outcomes for BMI SDS, for girls treated in Group 1 (12-weekly) (*n* = 26) and 24-weekly (*n* = 33), and boys treated in Group 1 (12-weekly) (*n* = 3) and 24-weekly (*n* = 3). BMI SDS was non-normally distributed and analyzed using the Mann–Whitney *U* test.

No differences in BMI progression across timepoints were observed for either regimen. Median BMI SDS was similar between treatment groups at baseline (HL difference −0.03, 95% CI −0.12 to 0.13; *P* & .93), after 1 year (−0.06, 95% CI −0.11 to 0.16; *P* & .70), and after 2 years (−0.08, 95% CI −0.18 to 0.15; *P* & .83) (Fig. 4A). Analyses stratified by sex confirmed no difference in girls (median 1.59 [12w, *n* = 26] vs 1.80 [24w, *n* = 33]; 95% CI −0.66 to 0.49; *P* = .90), and similarly in boys (median 1.08 [12w, *n* = 3] vs 1.84 [24w, *n* = 3]; 95% CI −1.35 to −0.39; *P* = .10), though interpretation is limited by the very small sample size (Fig. 4B).

Near-adult height data were available for a small subset of patients treated at one center (*n* & 12; Group 1 [12 weekly]: 8 girls, 1 boy; Group 2 [24 weekly]: 2 girls, 1 boy). In Group 1, near-adult height SDS in girls ranged from −1.3 to +1.2, with a median of −0.1 SDS, and was +1.8 in the single boy. In Group 2, near-adult height SDS in girls ranged from +0.13 to +0.33 and was +0.49 in the single boy. Due to the small sample size and single-center nature of these data, statistical comparisons between treatment regimens were not undertaken, but no differences between the regimens were apparent.

### Impact of treatment on pubertal progression

**Figure 5:**
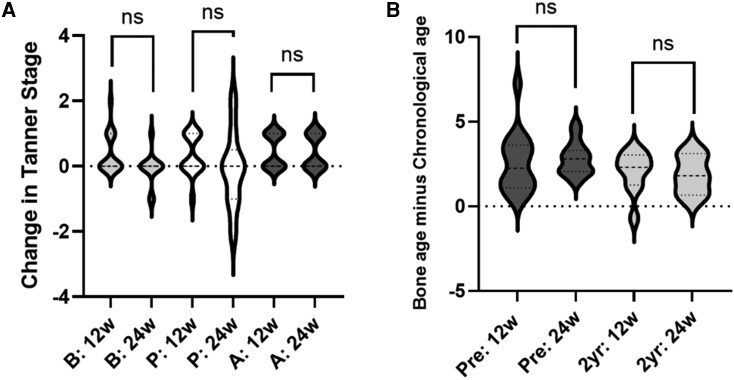
Progression in Tanner stage and BA–CA between baseline and 2 years. A Change in Tanner stage in girls during GnRHa treatment from baseline to 2 years shown for girls receiving 12-weekly or 24-weekly treatment, for breast development (B), pubic hair growth (P), and axillary hair growth (A). B BA–CA at baseline and posttreatment (2 years) in girls, comparing 12-weekly (12w) and 24-weekly (24w). Pre 12w (*n* = 16), Pre 24w (*n* = 15), 2 years 12w (*n* = 10), and 2 years 24w (*n* = 3). Tanner stage and BA–CA data were non-normally distributed and analyzed using the Mann–Whitney *U* test.

No differences in Tanner stage progression were observed between regimens in girls at 2 years (Fig. 5A). For breast development (12w: *n* = 24; 24w: *n* = 13), the HL difference was 0 stages (95% CI 0.0 to 1.0; *P* = .062). For pubic hair (12w: *n* = 16; 24w: *n* = 9), the difference was 0 stages (95% CI 0.0 to 1.0; *P* = .225). For axillary hair (12w: *n* = 16; 24w: *n* = 9), the difference was 0 stages (95% CI 0.0 to 0.5; *P* > .999). In boys, numbers were insufficient for comparison.

### Impact of treatment on bone maturation

Data for BA advancement were limited for boys in Group 2 (24-weekly) and at the 2-year timepoint, thus analyses were restricted to girls. Baseline BA–CA values were comparable between groups, with a median of 2.3 years in Group 1 (12-weekly) and 2.8 years in Group 2 (24-weekly), Hodges–Lehmann (HL) difference −0.42, 95% CI −1.37 to 0.68; *P* & .32. After 2 years of treatment, there had been no significant advancement in either regimen (Wilcoxon matched pairs signed rank test, Group 1 (12-weekly): *P* & .22, Group 2 (24-weekly): *P* > .99) and BA–CA remained comparable (2.4 vs 1.8 years; HL difference +0.37, 95% CI −0.95 to 1.71; *P* & .81) (Fig. 5B).

### Impact of treatment on biochemical markers of pubertal progression

**Figure 6:**
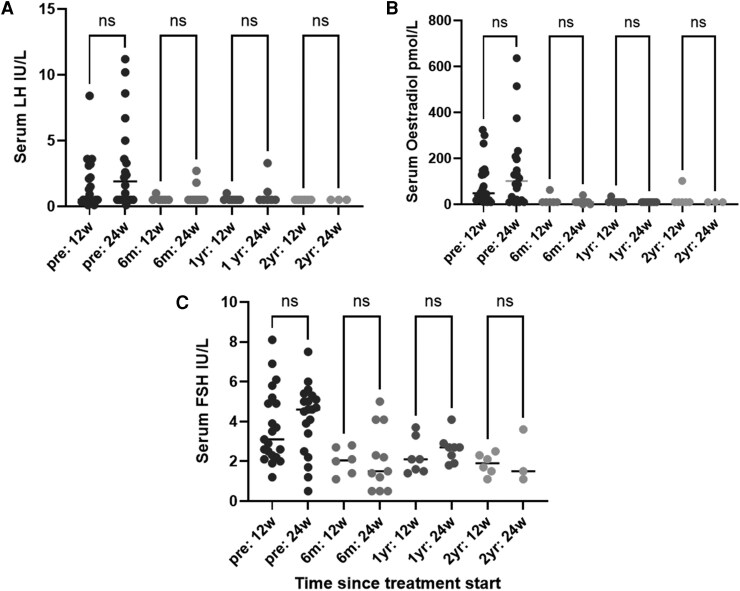
Endocrine biochemistry on GnRHa treatment. A Serum LH (IU/L) at pretreatment, 6 months, 1 year, and 2 years in girls (*n* = 23), comparing 12-weekly (12w) and 24-weekly (24w). HL differences (12w–24w) with 95% CIs: baseline −0.40 (−2.10 to 0.00; *P* = .11), 6 months 0.00 (−0.40 to 0.00; *P* = .81), 1 year 0.00 (−0.60 to 0.00; *P* = .48), 2 years 0.00 (0.00 to 0.00; *P* = 1.00). B Serum estradiol (pmol/L) at pretreatment, 6 months, 1 year, and 2 years in girls (*n* = 23), comparing 12-weekly (12w) and 24-weekly (24w). HL differences (12w–24w) with 95% CIs: baseline +18.0 (−31.3 to 148.3; *P* = .45), 6 months 0.00 (0.0 to 26.8; *P* = .32), 1 year 0.00 (0.0 to 0.0; *P* = .39), 2 years 0.00 (0.0 to 46.3; *P* = .64). C Serum FSH (IU/L) at pretreatment, 6 months, 1 year, and 2 years in girls (*n* = 23), comparing 12-weekly (12w) and 24-weekly (24w) regimens. HL differences (12w–24w) with 95% CIs: baseline −0.65 (−2.00 to 0.70; *P* = .38), 6 months +0.45 (−1.40 to 1.30; *P* = .84), 1 year −0.60 (−1.20 to 0.60; *P* = .27), 2 years +0.10 (−1.90 to 1.00; *P* = .90).

Following treatment initiation due to variations in clinical practice, consistent biochemical data were only available from RLH (*n* = 50); thus, patients from RVI and SCH were excluded from biochemical analyses. At baseline, LH, estradiol, and FSH values were comparable across regimens. Among girls, no significant differences were observed at any timepoint between Group 1 (12 weekly) and Group 2 (24 weekly) (Fig. 6A–6C). At 1 year, adequate suppression of LH (basal LH <1 IU/L prior to injection) was achieved in 86% (*n* = 6/7) of girls receiving 12-weekly treatment (95% CI 72-94) and 75% (*n* = 5/7) receiving 24 weekly (95% CI 62-85), with a risk difference of 11% (95% CI −6 to 27; *P* = .21). By 2 years, 100% of girls in both treatment groups demonstrated effective LH suppression (95% CI 82-100 and 78-100, respectively) (Fig. 6A). Due to limited data, differences between male treatment groups could not be assessed.

### Patient reported tolerance of GnRHa regimens

Adverse effects were assessed at each clinic visit. Both formulations were generally well tolerated, with most adverse events being mild and consistent with the known side-effect profile of GnRHa therapy. Given the small numbers, adverse event data are summarized descriptively without statistical analysis. The most frequently reported effects were injection site pain (12w: *n* & 7, 10%; 24w: *n* & 8, 8%), mood swings (12w: *n* & 5, 7%; 24w: *n* & 9, 9%), weight gain (12w: *n* & 4, 6%; 24w: *n* & 2, 2%), and abdominal pain (12w: *n* & 2, 3%; 24w: *n* & 3, 3%). A smaller number of participants reported headaches (12w: *n* & 3, 4%; 24w: *n* & 0). Two participants experienced both mood swings and weight gain (12w: *n* & 1, 1%; 24w: *n* & 1, 1%). Adverse events reported only once included: difficult behavior (12w), insomnia (12w), vaginal spotting (12w), constipation (24w), hot flushes (12w), vomiting (12w), clinginess (12w), concentration difficulties (24w), and increased appetite (24w). Of 13 participants who had experienced both the 12 and 24 weekly regimens, all 13 (100%) stated that they preferred the 24-weekly formulation, citing reduced appointment frequency, reduced disruption to schooling and reduced disruption to family life.

## Discussion

This retrospective, 3-center UK cohort study assessed the efficacy of 24-weekly 22.5 mg Decapeptyl SR compared to the standard 12-weekly 11.25 mg formulation in the treatment of CPP across a cohort of 247 patients. At presentation, the cohort demonstrated characteristic clinical features of CPP, including advanced Tanner staging for age, increased gonadotropin concentrations, and modestly elevated height and BMI SDS. There were no significant differences in baseline phenotypic features or age at presentation between patients at different centers, and the girl predominance (reflecting the known epidemiology of CPP) was consistent with previous studies (2, 3). The elevated BMI SDS observed aligns with existing literature suggesting an association between increased adiposity and earlier pubertal onset (25-28).

The primary objective of this study was to evaluate whether the less frequent 24-weekly formulation was as effective as the established 12-weekly regimen. Treatment outcomes, including LH suppression, HV, BA advancement, and Tanner staging, were assessed over a 2-year period. The 2 treatment regimen groups were comparable across all parameters at baseline (pretreatment). Between baseline and posttreatment analyses, exclusions were applied to ensure treatment groups remained standardized within a 2-week interval. The greater number of exclusions at SCH reflects a different clinical threshold for altering injection frequency, with clinicians more readily adjusting dosing when preinjection symptoms (eg, behavioral changes) suggested possible breakthrough effects. No significant differences were found in phenotypic outcomes between the 2 groups, supporting the conclusion that the 24-weekly formulation is comparably efficacious. Biochemical data were limited, reflecting the standard clinical practice of monitoring primarily based on symptoms rather than biochemical markers. By 2 years of treatment, all girls with available data had achieved LH suppression with a preinjection LH of <1 IU/L, although fewer in the 24-weekly group showed suppression at the 1-year mark. This modest delay may reflect the longer interval between dosing, but it did not translate to differences in clinical progression features including breast development, HV or BA advancement. This small subset of children did not demonstrate distinguishing clinical or biochemical features, including age at presentation or baseline gonadotropin concentrations, suggesting no obvious high-risk phenotype within this small subgroup.

Tolerability was high for both formulations, with no reported side effects beyond those typically associated with GnRH analogs, namely injection site pain, mood swings, and weight gain.

In addition to the practical and psychological benefits of fewer injections, there are likely to be meaningful financial implications. Precise NHS costs for Decapeptyl administration are determined by Healthcare Resource Group (HRG) codes, which vary by Trust, but estimated figures can be derived by combining published drug prices with typical outpatient procedure tariffs. The ex-VAT list price for Decapeptyl SR 11.25 mg is approximately £314, compared with £532 for the 22.5 mg preparation. When outpatient attendance and administration costs are included (around £250 in London settings), the estimated per-injection cost is £565 for the 11.25 mg regimen and £785 for the 22.5 mg regimen. Based on the median treatment durations observed in this study, this equates to a total cost of approximately £7345 for girls and £9605 for boys receiving the 11.25 mg regimen, compared with £3925 and £4710 for girls and boys, respectively, on the 22.5 mg regimen. This represents an estimated saving of around £3400 per girl patient and £4900 per boy patient when treated with the 24-weekly rather than the 12-weekly regimen. Thus, the 24-weekly formulation has the potential to halve the number of visits and reduce overall treatment costs per patient, alongside the benefits of lower healthcare resource utilization. For families, fewer hospital visits also minimize disruption to education and family life and limit the financial impact of lost income. Furthermore, a reduced frequency of injections is clinically relevant, as needled procedures remain one of the most common sources of fear and distress in children, with previous studies highlighting both the prevalence and impact of needle-related anxiety (29, 30). Although these estimates are based on NHS cost and clinical service structures, similar financial, patient, and healthcare resource benefits are likely to be realized across other healthcare systems internationally.

Although the 24-week regimen offers clear practical advantages, its longer dosing interval provides fewer routine opportunities for clinical assessment, which may delay recognition of inadequate suppression in a small number of patients. This underscores the importance of individualized monitoring and of counseling families to recognize and report signs of pubertal progression between doses. Given the comparable efficacy and tolerability of the 2 regimens demonstrated in this study, initiation of treatment with the 24-weekly Decapeptyl formulation is a reasonable first-line approach for most children with CPP. In those requiring additional clinical caution, such as younger children or boys, scheduling an early clinical and biochemical review after treatment initiation may be appropriate to confirm adequate suppression and guide ongoing management. This strategy preserves the benefits of reduced injection frequency while allowing timely identification of suboptimal response in higher-risk groups. Ongoing careful monitoring of growth and pubertal progression remains essential for all patients.

### Limitations

Although this study benefits from a large cohort, some limitations constrained the analysis due to its real-life pragmatic approach. The retrospective nature of data collection resulted in incomplete data capture across parameters and timepoints, limiting the ability to perform some subgroup analyses. Variable dosing practices between centers led to the exclusion of some patients from treatment outcome analysis due to incompatible regimens. Biochemical data were limited in some centers after treatment was initiated, reflecting clinical practice prioritizing clinical and auxological assessment of pubertal progression rather than hormonal concentrations. Additionally, variation in management approaches between clinicians, even within the same center, and the long study duration may have introduced inconsistencies in follow-up practices. Adherence data were not collected, so deviations between prescribed and actual injection timing could not be assessed. Total numbers were too small to allow meaningful analysis disaggregated by ethnic group. Finally, assessment of suppression in boys was limited by the small number of cases, a common constraint in CPP research due to the rarity of the condition in boys. Although male patients were included, comparative analyses in boys were limited by small numbers, and findings in this subgroup should be interpreted descriptively. Near-adult height data were available for only a small subset of patients from a single center, and the sample size was insufficient to permit intergroup comparisons between treatment regimens.

### Conclusion

In conclusion, this study supports the use of the 24-weekly 22.5 mg Decapeptyl SR regimen in the management of patients with CPP. It demonstrates similar efficacy and tolerability to the 12-weekly 11.25 mg formulation, while the reduced injection frequency offers tangible benefits for patients, families, and healthcare services, including lower treatment burden and potential cost savings. Further prospective, standardized studies with larger sample sizes and comprehensive biochemical data, particularly in male patients, are warranted to refine best practice.

## Data Availability

Some or all datasets generated during and/or analyzed during the current study are not publicly available but are available from the corresponding author on reasonable request.

## References

[1] Carel JC, Léger J. Clinical practice. Precocious puberty. N Engl J Med. 2008;358(22):2366‐2377. 18509122 10.1056/NEJMcp0800459

[2] Teilmann G, Pedersen CB, Jensen TK, Skakkebæk NE, Juul A. Prevalence and incidence of precocious pubertal development in Denmark: an epidemiologic study based on national registries. Pediatrics. 2005;116(6):1323‐1328. 16322154 10.1542/peds.2005-0012

[3] Bridges NA, Christopher JA, Hindmarsh PC, Brook CG. Sexual precocity: sex incidence and aetiology. Arch Dis Child. 1994;70(2):116‐118. 8129431 10.1136/adc.70.2.116 PMC1029712

[4] Cho AY, Shim YS, Lee HS, Hwang JS. Effect of gonadotropin-releasing hormone agonist monotherapy and combination therapy with growth hormone on final adult height in girls with central precocious puberty. Sci Rep. 2023;13(1):1264. 36690835 10.1038/s41598-023-28602-3 PMC9870989

[5] Mendle J, Ryan RM, McKone KMP. Age at menarche, depression, and antisocial behavior in adulthood. Pediatrics. 2018;141(1):e20171703. 29279324 10.1542/peds.2017-1703 PMC5744273

[6] Mishra GD, Pandeya N, Dobson AJ, et al Early menarche, nulliparity and the risk for premature and early natural menopause. Hum Reprod. 2017;32(3):679‐686. 28119483 10.1093/humrep/dew350 PMC5850221

[7] Brito VN, Canton APM, Seraphim CE, et al The congenital and acquired mechanisms implicated in the etiology of central precocious puberty. Endocr Rev. 2023;44(2):193‐221. 35930274 10.1210/endrev/bnac020 PMC9985412

[8] Canton APM, Tinano FR, Guasti L, et al Rare variants in the MECP2 gene in girls with central precocious puberty: a translational cohort study. Lancet Diabetes Endocrinol. 2023;11(8):545‐554. 37385287 10.1016/S2213-8587(23)00131-6 PMC7615084

[9] Choi KH, Chung SJ, Kang MJ, et al Boys with precocious or early puberty: incidence of pathological brain magnetic resonance imaging findings and factors related to newly developed brain lesions. Ann Pediatr Endocrinol Metab. 2013;18(4):183‐190. 24904875 10.6065/apem.2013.18.4.183 PMC4027080

[10] Vurallı D, Özön A, Gönç EN, Oğuz KK, Kandemir N, Alikaşifoğlu A. Gender-related differences in etiology of organic central precocious puberty. Turk J Pediatr. 2020;62(5):763‐769. 33108078 10.24953/turkjped.2020.05.007

[11] Kim YJ, Kwon A, Jung MK, et al Incidence and prevalence of central precocious puberty in Korea: an epidemiologic study based on a national database. J Pediatr. 2019;208:221‐228. 30857777 10.1016/j.jpeds.2018.12.022

[12] Hoskyns RB, Howard SR. Effects of the COVID-19 pandemic on the incidence of central precocious puberty; a narrative review. J Pediatr Endocrinol Metab. 2024;37(2):102‐109. 38097507 10.1515/jpem-2023-0507

[13] Zevin EL, Eugster EA. Central precocious puberty: a review of diagnosis, treatment, and outcomes. Lancet Child Adolesc Health. 2023;7(12):886‐896. 37973253 10.1016/S2352-4642(23)00237-7

[14] Hirsch HJ, Lahlou N, Gillis D, et al Free alpha-subunit is the most sensitive marker of gonadotropin recovery after treatment of central precocious puberty with the histrelin implant. J Clin Endocrinol Metab. 2010;95(6):2841‐2844. 20339028 10.1210/jc.2009-2078

[15] Lahlou N, Carel JC, Chaussain JL, Roger M. Pharmacokinetics and pharmacodynamics of GnRH agonists: clinical implications in pediatrics. J Pediatr Endocrinol Metab. 2000;13(Supplement):723‐737. 10969915 10.1515/jpem.2000.13.s1.723

[16] Breidbart E, Breidbart E, Ilkowitz J, et al Precocious puberty and GnRH analogs: current treatment practices and perspectives among US pediatric endocrinologists. Horm Res Paediatr. 2025;98(5):491‐502. 38718766 10.1159/000539011 PMC12416872

[17] Popovic J, Geffner ME, Rogol AD, et al Gonadotropin-releasing hormone analog therapies for children with central precocious puberty in the United States. Front Pediatr. 2022;10:968485. 36268040 10.3389/fped.2022.968485 PMC9577333

[18] Bangalore Krishna K, Fuqua JS, Rogol AD, et al Use of gonadotropin-releasing hormone analogs in children: update by an International Consortium. Horm Res Paediatr. 2019;91(6):357‐372. 31319416 10.1159/000501336

[19] Klein K, Yang J, Aisenberg J, et al Efficacy and safety of triptorelin 6-month formulation in patients with central precocious puberty. J Pediatr Endocrinol Metab. 2016;29(11):1241‐1248. 26887034 10.1515/jpem-2015-0376

[20] Yu X, Cheng X, Wei H, et al A phase 3, open-label, single-arm trial of the efficacy and safety of triptorelin 6-month formulation in Chinese children with central precocious puberty. Adv Ther. 2024;41(12):4537‐4556. 39412628 10.1007/s12325-024-02991-x PMC11550251

[21] Yoo E, Kim S, Jung HL, et al Impact of 6-month triptorelin formulation on predicted adult height and basal gonadotropin levels in patients with central precocious puberty. Front Endocrinol (Lausanne). 2023;14:1134977. 36875449 10.3389/fendo.2023.1134977 PMC9982112

[22] Howard S, Ghauri R, El-Khairi R. Guideline: GnRH Analogue Stimulation Testing to Investigate Precocious Puberty. British Society for Paediatric Endocrinology and Diabetes; 2021.

[23] Group WHOMGRS . WHO Child Growth Standards based on length/height, weight and age. Acta Paediatr Suppl. 2006;95:76‐85. 10.1111/j.1651-2227.2006.tb02378.x 16817681

[24] Klein KO, Miller BS, Mauras N. Unstimulated luteinizing hormone for assessment of suppression during treatment of central precocious puberty with 6-month subcutaneous leuprolide acetate: correlations with clinical response. Horm Res Paediatr. 2025;98(5):514‐523. 38684152 10.1159/000539110 PMC12416875

[25] Ong KK, Ahmed ML, Dunger DB. Lessons from large population studies on timing and tempo of puberty (secular trends and relation to body size): the European trend. Mol Cell Endocrinol. 2006;254-255:8‐12. 16757103 10.1016/j.mce.2006.04.018

[26] Kaplowitz PB, Slora EJ, Wasserman RC, Pedlow SE, Herman-Giddens ME. Earlier onset of puberty in girls: relation to increased body mass index and race. Pediatrics. 2001;108(2):347‐353. 11483799 10.1542/peds.108.2.347

[27] Biro FM, Khoury P, Morrison JA. Influence of obesity on timing of puberty. Int J Androl. 2006;29(1):272‐277. discussion 86–90. 16371114 10.1111/j.1365-2605.2005.00602.x

[28] Sørensen K, Aksglaede L, Petersen JH, Juul A. Recent changes in pubertal timing in healthy Danish boys: associations with body mass index. J Clin Endocrinol Metab. 2010;95(1):263‐270. 19926714 10.1210/jc.2009-1478

[29] Wright S, Yelland M, Heathcote K, Ng SK, Wright G. Fear of needles–nature and prevalence in general practice. Aust Fam Physician. 2009;38(3):172‐176. 19283260

[30] Kortesluoma RL, Nikkonen M. ‘I had this horrible pain’: the sources and causes of pain experiences in 4- to 11-year-old hospitalized children. J Child Health Care. 2004;8(3):210‐231. 15358886 10.1177/1367493504045822

